# Comparative Analysis of Fcγ and Complement Receptors Presence on Monocytes in Pulmonary Sarcoidosis and Tuberculosis

**DOI:** 10.3390/ijms24119713

**Published:** 2023-06-03

**Authors:** Marlena Typiak, Piotr Trzonkowski, Monika Skotarczak, Anna Dubaniewicz

**Affiliations:** 1 Department of General and Medical Biochemistry, Faculty of Biology, University of Gdansk, Wita Stwosza 59 St., 80-308 Gdansk, Poland; marlena.typiak@ug.edu.pl; 2 Department of Medical Immunology, Medical University of Gdansk, Debinki 7 St., 80-211 Gdansk, Poland; 3 1st Department of Radiology, Medical University of Gdansk, Mariana Smoluchowskiego 17 St., 80-214 Gdansk, Poland; 4 Department of Pulmonology, Medical University of Gdansk, Mariana Smoluchowskiego 17 St., 80-214 Gdansk, Poland

**Keywords:** sarcoidosis, tuberculosis, monocytes, Fc gamma receptors, IgG receptors, receptors, complement, phagocytosis, diagnosis, differential

## Abstract

Sarcoidosis (SA) is a granulomatous disorder, which mostly affects the lungs. Its clinical characteristics resemble tuberculosis (TB), but its treatment is different. The etiology of SA is unknown; however, mycobacterial antigens were proposed as environmental factors in its development. Due to previously revealed immunocomplexemia with mycobacterial antigens in the blood of our SA but not TB patients, and in the search for biomarkers for differential diagnosis of the two disorders, we studied the phagocytic activity of monocytes from both patients’ groups with flow cytometry. With the use of this method, we also analyzed the occurrence of receptors for IgG (FcγR) and complement components (CR) at the surface of these monocytes, responsible for phagocytosis of immunocomplexes. We revealed a higher phagocytic activity of monocytes in both disorders, but an increased frequency of monocytes with FcγRIII (CD16) and decreased with CR1 (CD35) receptor in the blood of SA vs. TB patients. With regard to our other genetic study on FcγRIII variants in SA and TB, this may account for the decreased clearance of immunocomplexes and different immune responses in the two diseases. Thus, the presented analysis not only sheds light on the pathomechanisms of SA and TB but may also support their differential diagnosis.

## 1. Introduction

Both sarcoidosis (SA) and tuberculosis (TB) are systemic granulomatous disorders that affect most frequently the respiratory system but are cured very differently. The common clinical, radiological, and histopathological characteristics of the two disorders often lead to misdiagnosis [1]. Treatment of sarcoidosis with antibiotics (such as those for TB) will not lead to the resolution of the disease. More importantly, treatment of tuberculosis with immunosuppressants (such as those for SA) leads to the exacerbation of the disease and can be very dangerous for the patient. Interestingly, glucocorticosteroid treatment for SA can lead some patients to the development of tuberculosis [2]. 

The etiologic factor of tuberculosis is clearly known (*Mycobacterium tuberculosis)*; however, we still lack knowledge of why the disease develops just in 5–10% of the infected individuals. The etiopathology of SA is yet to be clarified. Environmental (infectious and non-infectious), genetic factors, and autoimmunity have been studied as potential causes of sarcoidosis development [3,4]. Due to the confusingly similar characteristics of SA and TB [1], *M. tuberculosis* (Mtb) and its antigens, e.g., heat shock proteins (Mtb-hsp), have been suggested as the environmental component of SA development [5,6,7,8]. 

In contrast to TB, we reported significantly higher expression and level of Mtb-hsp70 than Mtb-hsp65 and Mtb-hsp16 in sarcoid tissues [9]. Higher expression of Mtb-hsp70 than Mtb-hsp16 and Mtb-hsp65 in sarcoid tissue could be caused via the sequestration of Mtb-hsp16 and Mtb-hsp65 antigens in the bound form, for example, in immune complexes (ICs) [7]. We have proven this concept and revealed significantly increased concentrations of ICs (consisting of immunoglobulin G (IgG) and especially Mtb-hsp16 or Mtb-hsp65) in sera from the same patients with SA [5,7,8]. Our results suggest that Mtb-hsp16 and Mtb-hsp65, being implicated in forming ICs, could be involved in an immune response against SA [6,10]. Moreover, the ability of ICs to induce a massive local inflammatory response was clearly established and could be responsible, e.g., for granuloma formation in both sarcoidosis and tuberculosis [11]. 

The shown antigenemia and immunocomplexemia were greater in SA than in TB patients, which could point to an aberrant IC clearance by phagocytes, especially in SA [8]. It could not be caused by a lowered number of mononuclear phagocytes, as the monocytes present in the blood of these SA patients were abundant and, in contrast to monocytes of TB patients, resistant to Mtb-hsp-induced apoptosis [10,12]. As we and others have also previously shown, the phagocytic activity of blood monocytes is significantly increased in SA patients compared to healthy individuals [13,14]. However, these monocytes have a decreased presence of complement receptor 1 (CR1 or CD35) and CR4 (CD11c); they have an abundance of all three classes of receptors for the Fc fragment of IgG (FcγRI-III or CD64/32/16) [13]. The balanced signaling via FcγR and CR receptors regulates the immune response, e.g., antigen presentation, phagocytosis, antibody-dependent cell-mediated cytotoxicity, regulation of cytokine expression, hydroxyl radicals production, and predispose subsets of human monocytes to become migratory dendritic cells (DCs) and/or macrophages [15,16,17]. However, FcγR and CR are crucial mostly for the removal of circulating immune complexes present in SA, whose increased concentration can result in chronic antigenemia and excessive lymphocyte proliferation. The answer to the mystery of immunocomplexemia in the blood of our SA patients in the abundant presence of FcγRI-III was a polymorphism of the *FCGR* genes that encode Fcγ receptors, which caused their aberrant function [18,19]. Changes in the function of monocytes in patients with TB infection are to be further clarified. As we have shown in our recent article, TB and SA patients differ in the polymorphism of *FCGR* genes [20]. This may cause discrepancies in the occurrence of FcγRs at the surface of their monocytes and enable a better differential diagnosis of SA and TB.

In the pursuit of etiopathogenetic distinction between SA and TB, and the search for biomarkers, that could help to make a differential diagnosis of these disorders, we have decided to study the rate of phagocytic activity of blood monocytes, as well as the presence of Fcγ and complement receptors on these cells also in patients with tuberculosis, and to compare it with results from SA patients and healthy individuals. Therefore, in this current article, we present a comparison of the number and percentage of FcγRI^+^ (CD64^+^), FcγRII^+^ (CD32^+^), FcγRIII^+^ (CD16^+^), CR1^+^ (CD35^+^), CR3^+^ (CD11b^+^), and CR4^+^ (CD11c^+^) peripheral blood monocytes in SA and TB patients, as well as TB patients and healthy individuals, which has never been published before.

## 2. Results

### 2.1. Presence of Monocytes in Peripheral Blood from SA Patients, TB Patients, and Healthy Control

**Figure 1:**
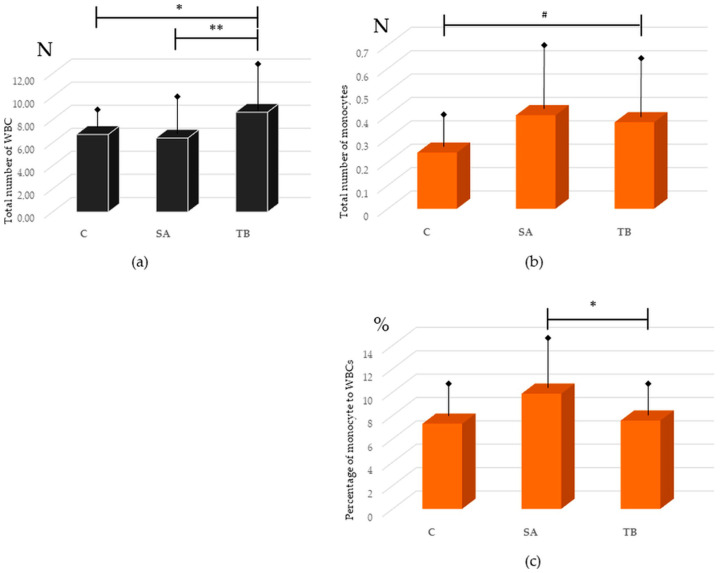
(**a**) Total number (N) of white blood cells (WBC) in the peripheral blood of patients with sarcoidosis (SA), patients with tuberculosis (TB), and healthy individuals (C); ** *p* = 0.003, * *p* = 0.028; (**b**) Total number of monocytes in the peripheral blood of healthy individuals, SA patients, and TB patients, # *p* = 0.04; (**c**) Percentage (%) of monocytes to all WBCs in peripheral blood of the studied groups; * *p* = 0.018. Significance of comparisons is presented for SA vs. TB and TB vs. C. Results are presented as mean (column) and standard deviation (arrow with hem).

We have revealed that the total number of white blood cells (WBC) was lower in the peripheral blood of SA versus TB patients (6.41 ± 2.63 vs. 8.65 ± 2.76, *p* = 0.003). We have also shown an increase in the total number of WBC in the blood of TB patients compared to healthy individuals (8.65 ± 2.76 vs. 6.72 ± 1.02, *p* = 0.028). There was no statistically significant difference between the total number of WBC in the blood of SA patients and the control group (Figure 1a).

The total number of monocytes was slightly higher in SA patients than in Cont. [13], as well as in TB patients compared to healthy individuals (0.40 ± 0.22 vs. 0.24 ± 0.09 and 0.37 ± 0.21 vs. 0.24 ± 0.09, *p* = 0.04 for both comparisons, respectively; Figure 1a). It did not differ between SA and TB patient groups (*p* > 0.05). The percentage of monocytes was significantly higher in SA than in the controls (Cont.) and TB patients (as shown previously for SA vs. Cont. [13]: 9.86 ± 3.42 vs. 7.29 ± 1.96, *p* = 0.005; for SA vs. TB: 9.86 ± 3.42 vs. 7.56 ± 1.76, *p* = 0.018). The percentage of monocytes did not differ between TB patients and the control group (*p* > 0.05; Figure 1b).

### 2.2. Percentage of Phagocyting Blood Monocytes in the Tested Groups

**Figure 2:**
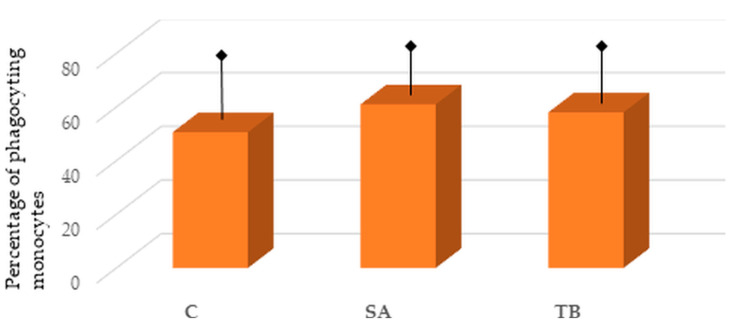
Percentage of phagocyting monocytes in the peripheral blood of patients with sarcoidosis (SA), patients with tuberculosis (TB), and healthy individuals (C). Comparisons are presented for SA vs. TB and TB vs. C (no significant differences). Results are presented as mean (column) and standard deviation (arrow with hem).

We have previously found a significantly higher phagocytic activity of monocytes in SA compared to the control group [13]. However, a slight elevation in the phagocytic activity of monocytes from SA patients than from TB patients can be seen; this difference did not reach a statistical significance (60.99 ± 10.43 vs. 58.03 ± 12.92, *p* > 0.05). Similarly, an increase in the phagocytic activity of monocytes, isolated from the blood of TB patients, can be observed compared to healthy individuals, but it is not significant (58.03 ± 12.92 vs. 50.60 ± 17.88, *p* > 0.05) (Figure 2).

### 2.3. Comparative Analysis of the Occurrence of FcγRI-III and CR1, 3, and 4 on Peripheral Blood Monocytes in All Tested Groups

Comparison of the presence of CD64^+^ and CD32^+^ monocytes revealed that the percentage and total number of CD32^+^ was higher than CD64^+^ cells in the control group, patients with SA, and TB patients. The analysis of CD32^+^ and CD16^+^ monocyte presence showed a higher percentage and total number of monocytes with CD32^+^ than CD16^+^ in controls and SA and TB patients. It is worth noticing that only in the SA patient group, the percentage and total number of CD16^+^ monocytes were significantly higher than that of CD64^+^ cells. Detailed results are presented in Table S1.

The percentage of CD35^+^ was significantly lower than CD11b^+^ and CD11c^+^ monocytes in the controls and SA patients. In the group of TB patients, the percentage of CD35^+^ was significantly lower than CD11b^+^ cells but not CD11c^+^ cells. The total number of CD35^+^ monocytes was significantly decreased compared to CD11b^+^ and CD11c^+^ cells only in the control group, whereas CD35^+^ cells were less frequent than CD11b^+^ cells only in patients with SA. The percentage of CD11b^+^ was significantly higher than CD11c^+^ monocytes only in the SA and TB patients and not the control group. There were no differences in the total number of CD11b^+^ and CD11c^+^ monocytes in the tested groups. Detailed results are presented in Table S1.

### 2.4. Comparative Analysis of the Percentage and Total Number of Monocytes with FcγRI-III and/or CR1, 3, and 4 in Peripheral Blood of SA and TB Patients 

**Figure 3:**
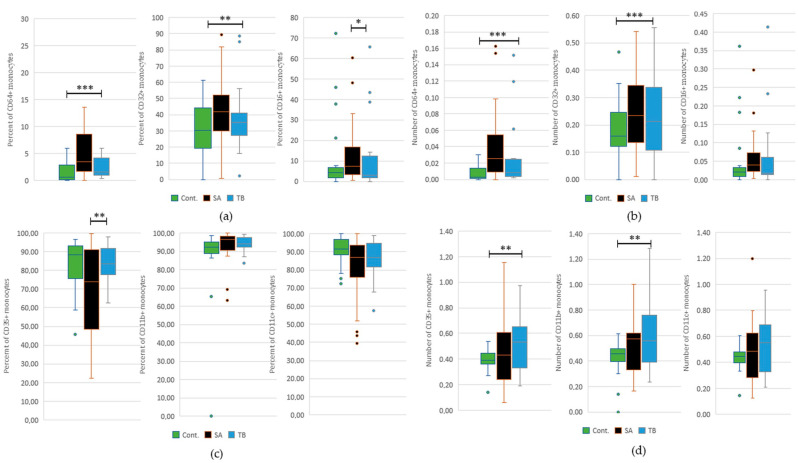
(**a**) Percentage of CD64^+^, CD32^+^, and CD16^+^ monocytes in the peripheral blood of patients with sarcoidosis (SA), patients with tuberculosis (TB), and healthy individuals (C), * *p* = 0.02, ** *p* = 0.008, *** *p* = 0.0005; (**b**) Total number of CD64^+^, CD32^+^, and CD16^+^ monocytes in the peripheral blood of the studied groups, *** *p* = 0.0005 for CD64^+^ and *p* = 0.0008 for CD32^+^; (**c**) Percentage of CD35^+^, CD11b^+^, and CD11c^+^ monocytes in the peripheral blood of the studied groups, ** *p* = 0.004; (**d**) Total number of CD35^+^, CD11b^+^, and CD11c^+^ monocytes in the peripheral blood of the studied groups, ** *p* = 0.01 for both comparisons. The graph shows the distribution of data in quartiles, highlighting the median and outliers. The percentage of a certain monocyte subpopulation stands for a fraction of monocytes with a particular receptor. Significance of comparisons is presented for SA vs. TB and TB vs. C. Comparisons between SA patients and healthy individuals were published previously [13].

This current study revealed a significantly increased percentage of CD16^+^ monocytes in SA patients in comparison to the patients suffering from TB. We have also shown a significant decrease in the percentage of CD35^+^ cells in the SA patient group than in TB patients (Figure 3). Detailed results are presented in Table S2.

**Figure 4:**
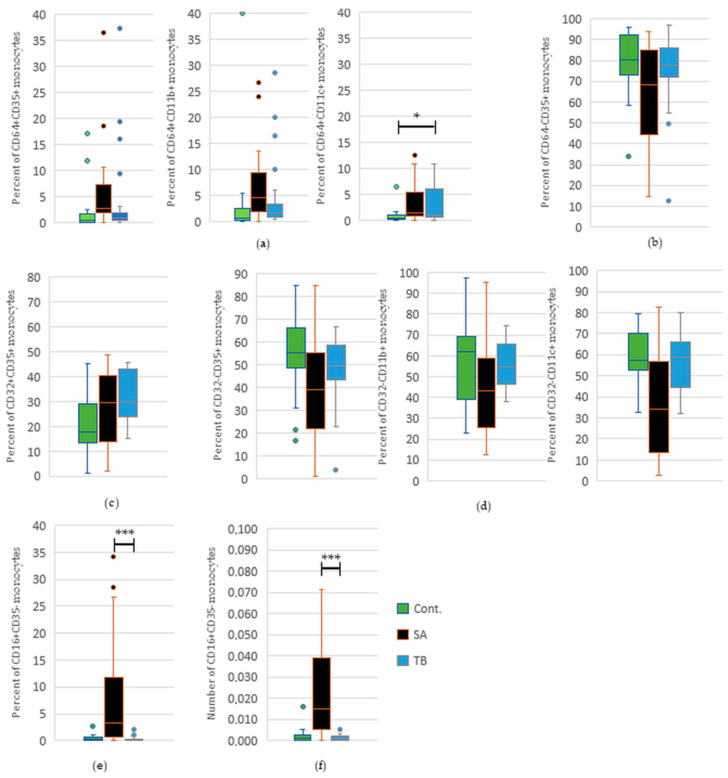
(**a**) Percentage of CD64+ monocytes with CD35, CD11b, or CD11c receptor, * *p* = 0.02; (**b**) Percentage of CD35+ monocytes lacking CD64 receptor; (**c**) Percentage of monocytes with CD32 and CD35 receptors; (**d**) Percentage of monocytes with CD35, CD11b, or CD11c, lacking CD32; (**e**) Percentage of CD16+ monocytes lacking CD35, *** *p* = 0.0002; (**f**) Total number of CD16+ monocytes lacking CD35, *** *p* = 0.0002. The graph shows the distribution of data in quartiles, highlighting the median or mean and outliers. The percentage of certain monocyte phenotypes stands for a fraction of monocytes with or without particular receptors. Due to the high number of possible phenotypes of the cells, only significant or nearly significant comparisons are presented in the figure. Significance of comparisons is presented for SA vs. TB and TB vs. C. Comparisons between SA patients and healthy individuals were published previously.

We have revealed a significant increase in the percentage and total number of CD16^+^CD35^−^ monocytes in the blood of SA versus TB patients. We have also found a decrease in the percentage of CD64^−^CD35^+^, CD32^−^CD11c^+^, and CD32^−^CD35^+^ cells in SA patients compared to patients with tuberculosis, but it did not reach statistical significance (Figure 4). Detailed results for significant or nearly significant comparisons are presented in Table S3.

### 2.5. Comparative Analysis of the Percentage and Total Number of Monocytes with FcγRI-III and/or CR1, 3, and 4 in Peripheral Blood of TB Patients and Healthy Controls

We have found a significant increase in the percentage and total number of CD64^+^ and CD32^+^ monocytes in the TB patient group in comparison to healthy individuals. We have also discovered a higher total number of CD35^+^ and CD11b^+^ cells in the peripheral blood of TB patients than in the control group (Figure 3). Detailed results are presented in Table S2.

We revealed a statistically significant increase in the percentage of CD64^+^CD11c^+^ monocytes in the blood of TB patients in comparison to healthy individuals. We have also found an increase in the percentage of CD64^+^CD35^+^, CD64^+^CD11b^+^, and CD32^+^CD35^+^ monocytes in TB patients versus the control group, but it did not reach statistical significance (Figure 4). Detailed results for significant or nearly significant comparisons are presented in Table S3.

## 3. Discussion

Monocytes, as well as other types of phagocytes, have a set of diverse cell surface receptors for recognition and reaction to extracellular environment changes, such as the appearance of immune complexes in response to antigen (Ag) load [21]. Fcγ and complement receptors cooperate for efficient clearance of antigens bound with opsonins (IgG and complement C proteins) from peripheral blood [16,22]. Both FcγR and CR are crucial for the phagocytosis of those particles and mediate immune cell activation, with the contribution of monocytes leading to reactions such as oxidative burst, cytotoxicity, and release of proinflammatory mediators [17,23]. Phagocyted antigens are cut into peptides and presented at the surface of antigen-presenting cells, e.g., monocytes, mainly to CD4^+^ and CD8^+^ T lymphocytes [24]. In most cases, this mechanism allows for IC clearance. However, while high antigen load, epitope spread, an impairment of the antigen degradation, or lowered Ag elimination occurs, persisting antigenemia and immunocomplexemia may cause excessive proliferation of lymphocytes and development of a granuloma, characteristic of both sarcoidosis and tuberculosis [25]. 

As we have shown in our previous studies, designed to find differences in the pathomechanisms of SA and TB development, patients with sarcoidosis showed a significantly increased level of Mtb-HSP-based immune complexes in their peripheral blood compared to patients with tuberculosis [8]. This has prompted us to find a reason for that discrepancy to support the differential diagnosis of the two disorders. First, we have focused on the function of peripheral blood mononuclear phagocytes (monocytes), which aberrated the number or function that could have led to this change.

Surprisingly, the monocytes in the blood of patients with sarcoidosis showed resistance to Mtb-HSP-induced apoptosis, and the cells from TB patients did not [10]. This is consistent with the currently shown significant increase in the percentage of peripheral blood monocytes in SA versus TB patients, which might not undergo apoptosis and persist on a high level in the blood of SA patients, and not patients with TB (no difference between TB and healthy control). This is also in accordance with the results of other authors, who have shown an increased frequency of monocyte occurrence in the blood and bronchoalveolar lavage fluid of patients with sarcoidosis in comparison to healthy controls [14]. However, to the best of our knowledge, no other analysis has been performed that compares monocyte frequency in SA and TB besides the currently presented one.

We have also checked the phagocytic activity of these monocytes, occurring abundantly in the blood of SA patients, and found an increase in this parameter for patients with SA in comparison to healthy individuals [13]. As we present in this current study, there was no significant difference in the phagocytic activity of monocytes between SA and TB patients (only slightly higher in SA). However, as there was a higher phagocytic activity of monocytes in the peripheral blood of patients with TB compared to healthy controls, it did not reach statistical significance. Thus, it is plausible that changes in the phagocytic activity of monocytes are present in both disorders, with the exception that in SA, the monocytes present with different characteristics regarding response to Mtb-HSP stimulation, which are part of ICs.

In order to find a reason for the elevated phagocytic activity of blood monocytes in patients with SA and TB (vs. control) and to determine the cause of failure to clear immune complexes from the blood of SA patients, but not patients with TB, we studied a total number and percentage of these cells with Fcγ and complement receptors. As we have previously shown, in the blood of patients with sarcoidosis, there is an overrepresentation of monocytes possessing all three classical classes of Fcγ receptors (FcγRI—CD64, FcγRII—CD32, and FcγRIII—CD16) and a decrease of these cells with CR1 (CD35) and CR4 (CD11c) [13]. In this current study, we show that the increased level of CD16^+^ monocytes in the blood of SA patients is not only significant in comparison to the healthy controls but also to the patients with TB. The same applies to the decrease in the percentage of CD35^+^ monocytes in the peripheral blood of SA patients versus patients with TB and healthy individuals. It is also accurate for the total number and percentage of CD16^+^CD35^−^ phenotype of monocytes.

The abundant presence of Fcγ receptors (especially FcγRIII—CD16) at the surface of peripheral blood monocytes of patients with sarcoidosis should have bound the immune complexes with Mtb-HSP and prevented the immunocomplexemia present in the blood of these patients. Because it did not, we turned to the analysis of functional polymorphism of the genes that encode Fcγ receptors and found in our SA patients a high representation of genetic variants translating to receptors with a lower ability to bind immune complexes [18,19]. We have deduced that the functional polymorphism of the *FCGR* genes, coding for Fcγ receptors, is the probable cause of the lowered binding and internalizing (via phagocytosis) of ICs by monocytes and immunocomplexemia present in the blood of our SA patients.

As we have shown in our recent paper, there is a difference in the representation of the variants of a gene-encoding CD16 (FcγRIII) receptor in monocytes (CD16A—FcγRIIIa), i.e., *FCGR3A*, in the genomes of SA and TB patients [20]. The analysis of a functional polymorphism of the *FCGR3A* gene revealed a significantly higher occurrence of the 158F allele and 158FF genotype, as well as a decreased occurrence of 158V and 158FV variants in SA patients (especially in Stage I of SA) versus patients with TB [20]. This can explain the presence of immunocomplexemia in the blood of our patients suffering from sarcoidosis, as the 158F variant of *FCGR3A* gene results in the forming of CD16A receptor with a lower IC binding ability than the receptor coded by the 158V variant of *FCGR3A* gene. Therefore, the CD16A receptor variant, present in the individuals who developed sarcoidosis, can be responsible for the lower clearance of ICs from the blood of these SA patients. Thus, we have revealed that the analysis of the percentage of blood monocytes with CD16 receptors at their surface could be a biomarker for a differential diagnosis of two clinically similar lung disorders: sarcoidosis and tuberculosis. Furthermore, the presented data support the knowledge of pathomechanisms leading to sarcoidosis and tuberculosis development, as they rely on the genetic predisposition of the affected individual with regard to the functional polymorphism of *FCGR3A* gene, translating to lower (in SA, especially in its initial stages) or higher (in TB) ability to bind and clear immune complexes from circulation. In the case of the initial, not so severe, stage I of SA, the lowered IC binding may lead to a decreased internalization of the immune complex with (e.g., mycobacterial) antigens, lower antigen processing, and presentation at the surface of monocytes, and trigger a lower activation of other immune cells and a smaller immune reaction than during TB development. Accordingly, the monocytes in the blood of TB patients with the high IC-binding version of the CD16A receptor may initiate an abundant immune response with T cell proliferation and the formation of vast numbers of granulomas [26], as seen in our patients with active TB.

The importance of CD16 receptor function in the development of sarcoidosis may be highlighted by the fact that the anti-sarcoidosis treatment with prednisone (an immune-suppressive glucocorticoid medication) and infliximab (IgG1 monoclonal antibody against tumor necrosis factor alpha) have been shown to downregulate pro-inflammatory intermediate (CD14^+^CD16^+^) and non-classical (CD14^−^CD16^++^) monocytes, and not the classical CD14^+^CD16^−^ monocytes. This may suggest an important role of the analysis of CD16 receptor presence on blood monocytes in determining disease activity and prognosis of the response to treatment in patients with sarcoidosis [27,28]. 

In this current study, we have also compared the total number and percentage of FcγR^+^ and CR^+^ monocytes in the peripheral blood of patients with tuberculosis and healthy individuals. We have revealed a significant increase in the percentage and total number of monocytes positive for CD64 and CD32 in TB patients versus the controls. It highlights the significant difference in the percentage of CD16^+^ monocytes between SA and TB patients since the fraction of these monocytes in TB and the controls does not differ and is significantly increased in sarcoidosis. The observation regarding the increased frequency of monocytes with CD64 (FcγRI) in the blood of TB patients vs. healthy individuals was also made by other Authors. They have obtained different results with regard to CD16 (FcγRIII) receptor presence since they showed an increase in the fraction of CD16^+^ monocytes in TB patients in comparison to healthy controls. However, they did not compare CD16^+^ monocyte presence in TB and SA patient groups [29]. 

The high amount of monocytes with CD64 and CD32 receptors at their surface enables them to effectively bind immune complexes. CD64 is also able to bind free IgG, not bound in an immune complex, whereas CD32 binds IgG only in an immune complex with an antigen, e.g., Mtb-HSP [16]. Our recent analysis focused on studying the functional polymorphism of *FCGR* genes, which encode IC-binding Fcγ receptors, including CD32 in monocytes (CD32A, CD32B, and CD32C). It has revealed a significant increase in the presence of the 57Q allele and 57XQ genotype of the *FCGR2C* gene (encoding CD32C) in TB patients vs. controls, which translates to the occurrence of a functional CD32C receptor, binding ICs effectively [20]. Therefore, the increased presence of highly functional CD32^+^ (CD32C^+^) monocytes in the peripheral blood of TB patients, compared to healthy individuals, may result in their increased phagocytic activity, antigen processing, and presentation to stimulate the immune response during the development of tuberculosis, including granuloma formation in the affected tissue.

We have also shown an increase in the total number of monocytes with complement receptors CD35 and CD11b in the blood of patients with TB in comparison to healthy individuals. The increase in the total number of monocytes positive for CD35 presence in the blood of TB patients versus healthy controls was not repeated in the analysis of CD35^+^ monocyte percentage. However, the decrease in the total number and percentage of CD35^+^ monocytes remained present in the blood of patients with SA, compared to TB, even if it is not statistically significant for the total number of cells. The decrease in CD35 receptors at the surface of monocytes in the blood of patients with sarcoidosis may result in a further decreased binding of circulating immune complexes and contribute to the immunocomplexemia present in our patients with sarcoidosis [8,17]. 

In the blood of patients suffering from tuberculosis, we have not only revealed the increased number of CD35^+^ and CD11b^+^ monocytes but also an increase in the percentage of CD64^+^CD11c^+^ cells (vs. healthy controls). It has been revealed that CD64 receptor expression on monocytes is upregulated within a few hours after infection and is correlated with the production of proinflammatory cytokines [21]. Additionally, in concordance with our results, other authors revealed a higher presence of CD11b and CD11c receptors at the surface of monocytes from TB patients [30]. These receptors can determine the transition of blood monocytes into tissue macrophages, migrating to the lungs, which is a major component of a granulomatous reaction during Mtb infection. It has been shown that different populations of monocytes transform into alveolar macrophages (CD11b^−^CD11c^+/high^), dendritic cells (CD11b^high^CD11c^+/high^), or small macrophages (CD11b^+/mid^CD11c^+/mid^) and that changes in the induction of these phenotypes occur at the early stage of Mtb infection [31]. Thus, the CD11b^+^ and CD11c^+^ monocytes, which frequency is increased in the blood of our TB patients, could transform into dendritic cells or small macrophages in the lungs of the affected individuals and take part in the process of granuloma formation. Moreover, CD11b was not only found to be important in phagocytosis but also in leukocyte recruitment, which may further contribute to the building of granulomas [17].

It is worth noticing that, unlike SA, in the blood of TB patients, there is an increase in the frequency of both FcγR^+^ and CR^+^ monocytes. This may transfer to a more efficient phagocytosis of ICs than in SA patients due to the strict cooperation of both types of receptors in IC binding. For example, to phagocytose iC3b-opsonized particles CD11b receptor must be activated by a signal from FcγR [17], such as CD64 or CD32 (both abundantly present on monocytes from TB patients).

In conclusion, the increased percentage of monocytes in the peripheral blood of SA versus TB patients, with their comparably high phagocytic activity, but different patterns of Fcγ and complement receptors with genetically coded different functional variants of CD16A (FcγRIIIa), could have resulted in different pathomechanisms and outcomes of SA and TB, even in the light of the suspected contribution of mycobacterial antigens in the development of sarcoidosis. The increase in the percentage of monocytes and in the occurrence of CD16 receptors on these cells (but with less functional genetically determined variants) might not be efficient enough to compensate for the deficiency of CD35 receptors on monocytes in sarcoidosis compared to TB. Together with a constant mycobacterial antigen load, revealed in our previous studies (reviewed in [5]), it may be a reason for the immunocomplexemia present in the blood of our SA patients and not in the patients with tuberculosis [8]. The persistent antigenemia and immunocomplexemia, with a decreased ability to process antigens and present them at the surface of monocytes, may contribute to the occurrence of the prolonged inflammatory response in SA patients, whereas the more effective IC-bound antigen engulfment (due to the increased presence of both FcγR and CR), processing, and presentation to T lymphocytes can translate into an exaggerated granulomatous immune response present in our patients with active tuberculosis. Thus, the analysis of CD16^+^ and CD35^+^ monocyte presence could support the differential diagnosis of the two very clinically similar disorders, as well as shed lighter on the discrepancies in the pathomechanisms of their development.

## 4. Materials and Methods

### 4.1. Study Participants

None of healthy controls, TB patients, or SA patients had a familial history of tuberculosis, sarcoidosis, or an autoimmune disease. None of the study participants were infected with human immunodeficiency virus (HIV) (Table 1).

#### 4.1.1. Patients with Sarcoidosis

The detailed characteristics of patients with sarcoidosis have been presented in our previous work [13]. Briefly, we included in this study 24 patients with newly diagnosed sarcoidosis (untreated) with mean age of 36.6 ± 8.8 years; range of 23–56 years; 10 women and 14 men. The diagnosis was made at the Pulmonology Hospital in Wejherowo, Poland, and was based on histological (scalenobiopsy of the lymph nodes), clinical, and radiological evidence. Diagnosis of Stage I of SA (bilateral hilar lymphadenopathy; 14 patients) and Stage II of SA (bilateral hilar lymphadenopathy and diffuse pulmonary infiltrations; 10 patients) was made after analysis of results of High-Resolution Computed Tomography. None of the patients had extrapulmonary sarcoidosis. An additional criterion for inclusion in the study group was a negative PPD (purified protein derivative) skin test (detecting TB). No acid-fast bacilli, fungi, or atypical cells were detected in microbiological and cytological examination (PCR and culture of *M. tuberculosis* strain) of the lymph nodes and sputum samples from the patients. 

#### 4.1.2. Patients with Tuberculosis

Twenty unrelated patients (mean age 45.9 ± 16.7 years; range 30–60 years; 5 women and 15 men) with newly detected active pulmonary tuberculosis were included in the study. A diagnosis of TB was made at the Pulmonology Hospital in Sopot and Wejherowo, Poland, and was established using standard clinical, radiographic, and bacteriological criteria. The patients were at a similar clinical stage and with similarly localized diseases on the initial chest radiographs (CXRs). No patient had extrapulmonary TB. The diagnosis of TB was confirmed via the demonstration of acid-fast bacilli in sputum smears and the positive sputum culture of *M. tuberculosis* strain. A positive PPD skin test was an additional criterion for inclusion in the study group. Patients were also classified according to their response to chemotherapy. Those who responded to the first line treatment (rifampin, isoniazid, ethambutol, pyrazinamide) were classified as drug responders.

#### 4.1.3. Healthy Controls

The detailed characteristics of the healthy individuals, included in this study, have been presented before [13]. Briefly, the control group consisted of twenty unrelated, healthy blood donors, who volunteered as study participants. The mean age of these individuals was 33.9 ± 11 years; range of 20–54 years. The group consisted of 10 women and 10 men, originating from the region of Gdansk (northern Poland). All control group members showed normal results of CXRs, blood, and serum analysis. They had no acid-fast bacilli in their sputum smears and in the sputum culture of *M. tuberculosis*. A PPD skin test was negative in all members of the control group. 

### 4.2. Methods

#### 4.2.1. Sample Collection and Preparation

Samples (15 mL) of fasting venous blood were collected into tubes with EDTA (Becton Dickinson Company, Franklin Lakes, NJ, USA). The number and percentage of certain subpopulations of peripheral blood mononuclear cells (PBMCs) were evaluated, and the expression of the tested FcγR and CR was assessed. The investigations were conducted prior to the treatment in both SA and TB groups of patients.

#### 4.2.2. Used Reagents and Antibodies

Single-use plastic equipment was supplied by NUNC (Thermo Fisher Scientific, Roskilde, Denmark). RPMI 1640 cell medium, fetal calf serum (FCS, inactivated), and other cell media were purchased from Gibco (Thermo Fisher Scientific, Waltham, MA, USA). The following monoclonal antibodies (mAb) were used in the flow cytometric study: anti-human CD14 (IgG2bκ PercPmAb, clone: MφP9) to mark peripheral blood monocytes; anti-human CD64 (IgG1κ FITCmAb, clone:10.1), anti-human CD32 (IgG2bκ FITCmAb, clone: FLI8.26), and anti-human CD16 (IgG1k FITCmAb, clone: 3G8) to analyze the presence of Fcγ receptors on these monocytes; anti-human CD35 (IgG1k PEmAb, clone:E11), anti-human CD11b (IgG1κ PEmAb, clone: ICRF44), and anti-human CD11c (IgG1k PEmAb, clone: B-ly6) to analyze the presence of complement receptors on these cells (all antibodies were purchased from BD Bioscience, PharMingen, Heidelberg, Germany). Appropriate surface isotype controls were implemented.

#### 4.2.3. PBMC Isolation

Mononuclear cells were isolated from the samples of peripheral blood with the use of Ficoll-Paque gradient centrifugation. Lymphocytes and monocytes (populations of PBMCs) were isolated together to mimic the conditions occurring in vivo, including possible cell-to-cell interactions between different subpopulations of PBMCs. PBMCs were subsequently analyzed via flow cytometry, as described previously in detail [10,11].

#### 4.2.4. Flow Cytometry

##### Analysis of Fcγ and Complement Receptor Presence at the Surface of CD14^+^ Monocytes

As we have described in our previous work, concerning the presence of FcγR and CR on monocytes in SA patients and healthy individuals [13], fresh PBMCs were vortexed and aliquoted into plastic tubes (12 × 75 mm) in the quantity of 2 × 10^5^ cells per tube. The cells were washed with staining buffer (phosphate-buffered saline (PBS) without Ca^2+^ or Mg^2+^, with 1% heat-inactivated FCS and 0.09% sodium acid). Surface staining of the monocytes was performed in the following combinations: CD14^+^CD64^+^, CD14^+^CD32^+^, CD14^+^CD16^+^, CD14^+^CD35^+^, CD14^+^CD11c^+^, CD14^+^CD11b^+^, CD14^+^CD64^+^CD35^+^, CD14^+^CD64^+^CD35^−^, CD14^+^CD64^−^CD35^+^, CD14^+^CD64^+^CD11b^+^, CD14^+^CD64^+^CD11b^−^, CD14^+^CD64^−^CD11b^+^, CD14^+^CD64^+^CD11c^+^, CD14^+^CD64^+^CD11c^−^, CD14^+^CD64^−^CD11c^+^, CD14^+^CD32^+^CD35^+^, CD14^+^CD32^+^CD35^−^, CD14^+^CD32^−^CD35^+^, CD14^+^CD32^+^CD11b^+^, CD14^+^CD32^+^CD11b^−^, CD14^+^CD32^−^CD11b^+^, CD14^+^CD32^+^CD11c^+^, CD14^+^CD32^+^CD11c^−^, CD14^+^CD32^−^CD11c^+^, CD14^+^CD16^+^CD35^+^, CD14^+^CD16^+^CD35^−^, CD14^+^CD16^−^CD35^+^, CD14^+^CD16^+^CD11b^+^, CD14^+^CD16^+^CD11b^−^, CD14^+^CD16^−^CD11b^+^, CD14^+^CD16^+^CD11c^+^, CD14^+^CD16^+^CD11c^−^, and CD14^+^CD16^−^CD11c^+^ with appropriate isotype controls. The samples were incubated for 30 min, in the dark, at room temperature. They were subsequently washed and fixed (4% paraformaldehyde (Sigma-Aldrich (MERCK), Rahway, NJ, USA) in PBS without Ca^2+^ and Mg^2+^). Fresh PBMCs were then analyzed with flow cytometry. Listmodes were acquired on Epics XL flow cytometer (Beckman Coulter, Brea, CA, USA) and using Winlist, software version 5.0 (as described in [10]). 

##### Analysis of the Phagocytic Activity of Monocytes

The assessment of the level of phagocytic activity of peripheral blood monocytes was performed with the use of Phagotest kit (ORPEGEN Pharma, Heidelberg, Germany), strictly according to the manufacturer’s instructions. The test enables us to assess phagocytic activity of monocytes in whole blood samples on the basis of measuring the ingestion of fluorescein (FITC) labeled opsonized *Escherichia coli*. It measures the percentage of phagocytes which have ingested bacteria. The fluorescence of not-internalized FITC-labeled *E. coli* is quenched before measurement.

#### 4.2.5. Statistical Analysis

Statistical comparisons between the three study groups were performed using the STATISTICA for Windows v. 8.0 software (TIBCO Software, Stanford Research Park, Palo Alto, CA, USA). An initial analysis with the W Shapiro–Wilk test was made to assess if parametric or non-parametric statistical tests should be implemented to analyze the obtained data. Afterward, we used the ANOVA Kruskal–Wallis test, the two-tail paired T-test for dependent or independent samples, and the Mann–Whitney U test to analyze the differences in the studied parameters between the tested groups. The results were presented as mean (±standard deviation) or median (with range), with the *p* value ≤ 0.05. Only significant differences were presented in full form.

## Figures and Tables

**Table 1:** Characteristics of patients suffering from sarcoidosis (SA), pulmonary tuberculosis (TB), and healthy controls (C). Percentages (%) of individuals with specific characteristics are given in parentheses.

		SA *n* = 24 (%)	TB *n* = 20 (%)	C *n* = 20 (%)
Age (years)	Mean ± SD	36.6 ± 8.8	45.9 ± 16.7	33.9 ± 11
	Range	23–56	30–60	20–54
Sex	Female	10 (42)	5 (25)	10 (50)
	Male	14 (58)	15 (75)	10 (50)
Smokers		20 (83)	16 (81)	15 (75)
BCG vaccinated		24 (100)	20 (100)	20 (100)
Positive PPD skin test		0	20 (100)	0
Löfgren’s syndrome		0	0	0
Relapse		0	0	0
Symptoms	Cough	11 (46)	18 (90)	0
	Dyspnea	0	5 (25)	0
	Increased body temperature	4 (17)	13 (65)	0
	Night sweats	0	10 (50)	0

BCG—Bacillus Calmette–Guérin vaccine against tuberculosis; SD—standard deviation; a purified protein derivative (PPD) skin test is a method used to diagnose TB infection.

## Data Availability

The data presented in this study are available in this current article and Supplementary Material.

## Supplementary Materials

The supporting information can be downloaded at: https://www.mdpi.com/article/10.3390/ijms24119713/s1.
