# Supplementary material for: Comparative Analysis of Fcγ and Complement Receptors Presence on Monocytes in Pulmonary Sarcoidosis and Tuberculosis
Source: Int J Mol Sci. 2023 Jun 3;24(11):9713. doi: 10.3390/ijms24119713 (PMC10253566; doi:10.3390/ijms24119713)
Supplement: Supplementary file 1 [file ijms-24-09713-s001.zip › ijms-2395593-supplementary.pdf]

Supplementary Materials to article:

Comparative Analysis of Fcγ and Complement Receptors Presence on Monocytes in Pulmonary Sarcoidosis and Tuberculosis

Marlena Typiak [1], Piotr Trzonkowski [2] Monika Skotarczak [3] and Anna Dubaniewicz [4,*]

1 Department of General and Medical Biochemistry, Faculty of Biology, University of Gdansk, Wita Stwosza 59 St., 80-308, Gdansk, Poland; marlena.typiak@ug.edu.pl

2 Department of Medical Immunology, Medical University of Gdansk, Debinki 7 St, 80-211, Gdansk, Poland

3 1st Department of Radiology, Medical University of Gdansk, Mariana Smoluchowskiego 17 St., 80-214, Gdansk, Poland

4 Department of Pulmonology, Medical University of Gdansk, Mariana Smoluchowskiego 17 St., 80-214, Gdansk, Poland

* Correspondence: aduban@gumed.edu.pl

Table S1. Percentage (%) and total number (N) of $CD64^{+}$, $CD32^{+}$, $CD16^{+}$ and $CD35^{+}$, $CD11b^{+}$, $CD11c^{+}$ monocytes in the peripheral blood of patients with sarcoidosis (SA), patients with tuberculosis (TB) and healthy controls (Cont.) - comparisons between monocytes with particular receptors. The provided p values are for comparisons made with t Student statistical test.

The tested parameter		CD35	CD11b	CD11c	p value
Cont.	(%)		90.71 ± 13.76	89.08 ± 9.44	0.45
TB	(%)		93.90 ± 6.18	87.29 ± 9.49	0.0001
SA	(%)		87.87 ± 15.63	78.96 ± 21.18	0.006
Cont.	(%)	81.23 ± 14.34	90.71 ± 13.76		0.0003
TB	(%)	84.56 ± 8.27	93.90 ± 6.18		0.0001
SA	(%)	69.39 ± 23.30	87.87 ± 15.63		0.0001
Cont.	(%)	81.23 ± 14.34		89.08 ± 9.44	0.0006
TB	(%)	84.56 ± 8.27		87.29 ± 9.49	0.14
SA	(%)	69.39 ± 23.30		78.96 ± 21.18	0.01
Cont.	(N)		0.44 ± 0.11	0.43 ± 0.09	0.53
TB	(N)		0.54 ± 0.19	0.50 ± 0.19	0.37
SA	(N)		0.55 ± 0.30	0.48 ± 0.25	0.13
Cont.	(N)	0.39 ± 0.09	0.44 ± 0.11		0.006
TB	(N)	0.49 ± 0.18	0.54 ± 0.19		0.19
SA	(N)	0.44 ± 0.25	0.55 ± 0.30		0.02
Cont.	(N)	0.39 ± 0.09		0.43 ± 0.09	0.01
TB	(N)	0.49 ± 0.18		0.50 ± 0.19	0.69
SA	(N)	0.44 ± 0.25		0.48 ± 0.25	0.34

Table S1 continued					
The tested parameter		CD64	CD32	CD16	p value
Cont.	(%)	4.30 ± 11.97	34.16 ± 17.58		0.0001
TB	(%)	7.71 ± 12.99	43.11 ± 17.51		0.0001
SA	(%)	5.86 ± 6.89	41.85 ± 20.05		0.0001
Cont.	(%)	4.30 ± 11.97		9.09 ± 16.37	0.07
TB	(%)	7.71 ± 12.99		13.32 ± 20.98	0.13
SA	(%)	5.86 ± 6.89		14.65 ± 17.29	0.0002
Cont.	(%)		34.16 ± 17.58	9.09 ± 16.37	0.0001
TB	(%)		43.11 ± 17.51	13.32 ± 20.98	0.0001
SA	(%)		41.85 ± 20.05	14.65 ± 17.29	0.0001
Cont.	(N)	0.02 ± 0.07	0.17 ± 0.10		0.0001
TB	(N)	0.05 ± 0.09	0.25 ± 0.14		0.0001
SA	(N)	0.03 ± 0.04	0.26 ± 0.18		0.0001
Cont.	(N)	0.02 ± 0.07		0.05 ± 0.10	0.06
TB	(N)	0.05 ± 0.09		0.05 ± 0.10	0.92
SA	(N)	0.03 ± 0.04		0.05 ± 0.07	0.0475
Cont.	(N)		0.17 ± 0.10	0.05 ± 0.10	0.0001
TB	(N)		0.25 ± 0.14	0.05 ± 0.10	0.0001
SA	(N)		0.26 ± 0.18	0.05 ± 0.07	0.0001

Table S2. Percentage (%) and total number (N) of $CD64^+$, $CD32^+$, $CD16^+$ and $CD35^+$, $CD11b^+$, $CD11c^+$ monocytes in the peripheral blood of patients with sarcoidosis (SA), patients with tuberculosis (TB) and healthy controls (Cont.) - comparisons between the tested groups. The provided p values are for comparisons made with the Mann-Whitney U test.

	Study group						p value for		
	Cont.		SA		TB				
Parameter	median	min-max	median	min-max	median	min-max	SA vs. Cont.	SA vs. TB	TB vs. Cont.
% $CD64^+$	0.50	0-62.85	2.95	0-37.97	1.15	0-50.07	0.0001	0.08	0.0005
% $CD32^+$	31.60	0-70.50	41.62	0.47-97.58	40.75	0-90.29	0.02	0.84	0.008
% $CD16^+$	3.41	0-72.25	6.75	0.30-62.64	2.62	0-72.17	0.002	0.02	0.78
N $CD64^+$	0.00	0-0.37	0.02	0-0.22	0.007	0-0.43	0.0001	0.09	0.0005
N $CD32^+$	0.15	0-0.46	0.25	0.001-1.17	0.21	0-0.56	0.002	0.86	0.0008
N $CD16^+$	0.01	0-0.41	0.027	0.0006-0.30	0.015	0-0.46	0.12	0.46	0.35
% $CD35^+$	87.16	34.62-99.06	74.06	12.50-99.64	85.61	62.59-98.05	0.01	0.004	0.64
% $CD11b^+$	93.00	0-100	94.86	34.26-100	95.55	72.02-100	0.72	0.11	0.09
% $CD11c^+$	91.40	60.08-100	86.63	9.91-100	89.41	63.67-99.61	0.008	0.11	0.24
N $CD35^+$	0.40	0.13-0.60	0.43	0.04-1.18	0.5	0.21-0.84	0.44	0.1	0.01
N $CD11b^+$	0.47	0-0.61	0.53	0.12-1.49	0.55	0.23-0.91	0.047	0.68	0.01
N $CD11c^+$	0.44	0.14-0.60	0.48	0.06-1.20	0.51	0.20-0.87	0.19	0.31	0.1

Table S3. Percentage (%) and total number (N) of monocytes with or without CD64, CD32, CD16 and CD35, CD11b, CD11c receptors, in the peripheral blood of patients with sarcoidosis (SA), patients with tuberculosis (TB) and healthy controls (Cont.) - comparisons between the tested groups. Due to the high number of possible phenotypes of the cells, only significant or nearly significant comparisons are presented in the table.

	Study group						p value for			
	Cont.		SA		TB					
Parameter	median/mean	min-max/SD	median/mean	min-max/SD	median/mean	min-max/SD	SA vs. Cont.	SA vs. TB	TB vs. Cont.	test
% $CD64^{+}CD35^{+}$	0.34	0 - 17.70	2.6	0 - 0.16	0.96	0 - 37.21	0.002	0.15	0.06	U Mann-Whitney
% $CD64^{-}$ $CD35^{+}$	80.18	33.90 - 95.74	68.22	14.81 - 93.75	79.66	12.52 - 95.83	0.03	0.09	0.91	U Mann-Whitney
% $CD64^{+}CD11b^{+}$	0.64	0 - 40.03	4.6	0 - 26.58	1.26	0.41 - 28.57	0.003	0.14	0.07	U Mann-Whitney
% $CD64^{+}CD11c^{+}$	0.42	0 - 57.14	1.47	0 - 12.50	1.62	0 - 14.50	0.01	0.9	0.02	U Mann-Whitney
% $CD32^{+}CD35^{+}$	23.91	17.57	27.19	14.47	34.42	13.84	0.53	0.16	0.07	t Student
% $CD32^{-}$ $CD35^{+}$	55.1	16.67 - 84.85	38.84	0.91 - 84.88	52.67	3.99 - 66.71	0.03	0.08	0.32	U Mann-Whitney
% $CD32^{-}$ $CD11b^{+}$	57.77	21.14	43.59	21.92	55.5	21.23	0.047	0.1	0.56	t Student
% $CD32^{-}$ $CD11c^{+}$	60.39	15.7	35.71	25.22	51.32	22.71	0.0009	0.07	0.2	t Student
% $CD16^{+}$ $CD35^{-}$	0.23	0 - 2.68	3.23	0 - 34.10	0.19	0 - 1.99	0.0001	0.0002	0.62	U Mann-Whitney
N $CD16^{+}CD35^{-}$	0.001	0 - 0.02	0.01	0 - 0.18	0.001	0 - 0.006	0.0001	0.0002	0.9	U Mann-Whitney
